# Performance error-related activity in monkey striatum during social interactions

**DOI:** 10.1038/srep37199

**Published:** 2016-11-16

**Authors:** Raymundo Báez-Mendoza, Wolfram Schultz

**Affiliations:** 1 Department of Physiology, Development and Neuroscience, University of Cambridge, Cambridge, CB2 3DY, UK

## Abstract

Monitoring our performance is fundamental to motor control while monitoring other’s performance is fundamental to social coordination. The striatum is hypothesized to play a role in action selection, action initiation, and action parsing, but we know little of its role in performance monitoring. Furthermore, the striatum contains neurons that respond to own and other’s actions. Therefore, we asked if striatal neurons signal own and conspecific’s performance errors. Two macaque monkeys sitting across a touch-sensitive table in plain view of each other took turns performing a simple motor task to obtain juice rewards while we recorded single striatal neurons from one monkey at a time. Both monkeys made more errors after individually making an error but made fewer errors after a conspecific error. Thus, monkeys’ behavior was influenced by their own and their conspecific’s past behavior. A population of striatal neurons responded to own and conspecific’s performance errors independently of a negative reward prediction error signal. Overall, these data suggest that monkeys are influenced by social errors and that striatal neurons signal performance errors. These signals might be important for social coordination, observational learning and adjusting to an ever-changing social landscape.

Learning to scull a boat requires balance and motor coordination. Learning this skill, or any other motor task, requires an online evaluation of performance. This process is called performance monitoring, which refers to “a set of cognitive and affective functions determining whether adaptive control is needed and, if so, which type and magnitude are required”^1^. A vital component of this comparison processes is a neuronal signal related to error commission. Primate anterior cingulate cortex contains such a signal^2^,^3^,^4^,^5^,^6^. The activity of this area along with the basal ganglia is hypothesized to aid action selection and motor adaptation^7^,^8^.

Actions may also occur in coordination with others, however. For example rowing in a boat with seven other people not only requires individual balance and motor coordination but necessitates motor coordination among the crew. Thus, this activity requires monitoring own and other’s performance. A neuronal correlate of this latter cognitive function has been found in the activity of medial frontal cortex neurons^9^. Neurons in this area signalled not only own errors but also other’s performance errors.

However, the role of the basal ganglia, and the striatum, in particular, has not been studied in relation to observational performance monitoring. We have previously shown that the striatum contains neurons that encode whose action lead to an own reward^10^. Thus, there exists the possibility that the activity of striatal neurons can detect own and conspecific errors. To substantiate this view, it is also vital to test behaviourally if the animals are sensitive to own and conspecific errors. To test both hypotheses, we trained two monkeys, sitting face to face, to take turns completing a motor task in which we manipulated the payoff matrix to avoid confounding error-related activity with reward-related activity while recording the activity of striatal neurons in one monkey at a time (Fig. 1A).

## Results

### Behaviour

**Figure 1:**
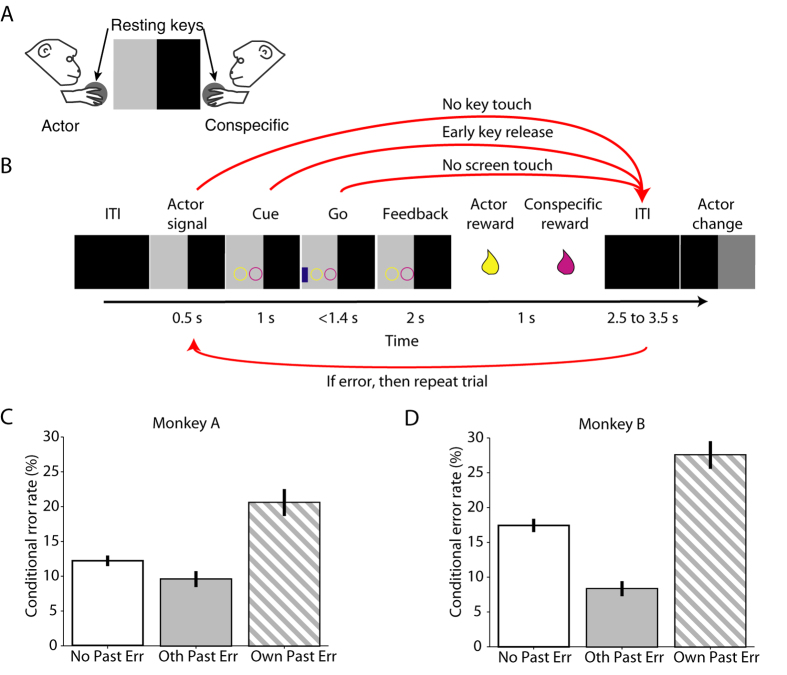
Behavioral task and associated errors. (**A**) Experimental setup. Two monkeys sat opposite each other at a horizontal computer touch monitor, each contacting a resting key. On each trial, light grey and black backgrounds indicated actor and conspecific roles to the respective animals. (**B**) Behavioral task timeline. The actor could make 3 different errors, signaled with red arrows: No key touch, the actor did not touch resting key. Early key release, the actor released the key before go signal appeared. No screen touch, the actor did not touch the area around the go signal in the touchscreen. The shape of the cues predicted absence (square) or presence (circle) of reward for each animal. We tested the following conditions: reward for neither, own reward only, conspecific’s reward only, and reward for both. (**C,D**) Mean conditional error rate following no past errors from either monkey (No Past Err; white), following conspecific’s past error (Oth Past Err; grey) or following own past error (Own Past Err; diagonal stripes) for monkeys A and B, respectively. Error bars show SEM.

A trial started when half of the screen closest to the acting monkey turned grey to indicate that it was its turn. To complete a trial the acting monkey reached the touch sensitive screen placed horizontally between the animals after a go-signal. The actor could commit three different types of errors (Fig. 1B): (1) not touching the resting key, (2) releasing the resting key before the onset of the go-signal, (3) not touching the touchscreen before timeout (1.4 s). If the actor committed any of these errors the screen turned black and a timeout of 5 to 7 s (planned trial time + 0.5 s) ensued, the animals did not switch roles, and the trial was reinitiated. Importantly, the animals did not switch roles until a correct trial was performed.

We explored the influence of previous behavioral errors by the actor and its conspecific on the actor’s behavior by estimating conditional error rates, which we defined as the percentage of errors committed in the current trial parsed by what had occurred in the previous trial. This metric allowed us to measure the behavioural impact of previous own errors as well as conspecific errors on the actor’s behaviour. For this analysis, we removed all correction trials, i.e. trials immediately following an error and averaged across neuronal recording sessions (n = 129). If the actor committed an error in the previous trial the probability that the actor also made an error in the current trial increased significantly in both animals. In monkey A the conditional error rate rose from 12.2 ± 0.07% (mean ± SEM) to 20.5 ± 1.9% following an own error and in monkey B it rose from 17.4 ± 0.09% to 27.5 ± 1.9% (Fig. 1C, D; t(128) = −5.29, p = 4.9 × 10-7; t(128) = −5.90, p = 2.9 × 10-8, respectively, paired t-test). By contrast, if the conspecific had committed an error in the previous trial the error rate diminished significantly for both animals, (by 2.6% and 9% in monkeys A and B respectively; t(128) = 2.55, p = 0.012; t(128) = 6.64, p = 8.04 × 10-10). The effect of the conspecific’s error on its partner’s behaviour is remarkable given that there was an intervening correct trial.

To further capture the effect of past own and conspecific errors on own error rate we used logistic regression. Own past errors and conspecific errors had a significant effect on the conditional error rate in both monkeys (Monkey A, own errors: t(13314) = 29.9, p = 4 × 10-197, conspecific errors: t(13314) = −5.17, p = 2 × 10-7; monkey B, own errors: t(14028) = 31.9, p = 1 × 10-223, conspecific errors: t(14028) = −8.3, p = 4 × 10-17). The effect of past errors on current errors was similar in both animals, therefore we validated each regression model by testing it with the choice data from the other monkey. The accuracy of the models to predict the errors of the other monkey were 75.5% and 80.5%; and the area under the receiver operating characteristic (AUROC), a measure of the model’s accuracy, was 0.61 for both models. To test the robustness of the logistic model against possible dependencies in the data we estimated the 95% confidence intervals (CI) of the odds ratio (OR) for each predictor using bootstrap with 2,000 iterations. This method removes the temporal structure in the data by sampling with replacement from the observed trials and then estimating the OR for each predictor using logistic regression. We found that a conspecific error had an OR in monkey A of 0.57 (0.45, 0.70; 95% CI) and in monkey B of 0.40 (0.31, 0.51; 95% CI), while an own error had an OR in monkey A of 4.35 (3.83, 4.93; 95% CI) and in monkey B of 3.97 (3.57, 4.43; 95% CI). In conclusion, all confidence intervals estimated with bootstrap did not cross 1, showing that the predictors were robust against any possible lack of independence in the error data. Overall, these data suggest that both monkeys’ performance was biased by own and conspecific’s previous performance.

It is well established that predicted reward in the current trial diminishes error rate while predicted reward absence increases it^11^. Both monkeys committed less errors in own reward trials than in own no-reward trials (reward presence vs. reward absence: 17.7% ± 1.2% vs. 6.6% ± 0.6%; 22.9% ± 1.14% vs. 4.8% ± 0.41%, for monkeys A and B respectively; both p < 4.6 × 10-13, t-test). Hence, we tested if the presence of own reward during conspecific’s error trials modified the conditional error rate following a conspecific’s error. We did this by including a term capturing conspecific error trials with own reward presence to the logistic regression. We found that own reward presence during conspecific’s error trials had no effect on the error rate (t(13314) = 0.54, p = 0.58 and t(14028) = −0.504, p = 0.61; monkeys A and B, respectively). The reward rate, i.e. the number of rewards received per unit of time, might alternatively explain the decrease in errors following a conspecific’s error. Indeed, we found that following a conspecific’s error the own reward rate diminished significantly from 4.48 ± 0.04 (mean ± SEM) rewards per minute to 3.88 ± 0.07 in Monkey A and from 4.43 ± 0.04 rewards per minute to 3.76 ± 0.08 in Monkey B (both p < 1 × 10-11, t-test).

To test if the animals lost motivation to participate in the task we compared the error rate in the first half of a session to the error rate in the second half of a session. We observed that as the animals progressed through the day their error rates increased from 11% to 14% in monkey A and from 16% to 23% in monkey B. The increases in error rates were significant in both animals (p < 0.02 and p < 0.008, in monkeys A and B respectively, paired t-test), indicating that they lost motivation as they became increasingly satiated during task performance.

Overall these results suggest that monkeys’ behavior was influenced not only by their past behavior but also by their conspecifics past behavior. Interestingly, the influence of the conspecific’s past behavior on current behavior did not depend on the presence of reward, but rather on a change of reward rate.

### Neurons

#### Error-coding neurons

For the analysis of neuronal responses to the trial outcome, we only included neurons that were recorded while both animals made at least three errors each. Although some neurons were recorded with only 6 errors, the average number of errors made per session was 29 ± 1.4 (mean ± SEM). The neuronal database comprised 165 single neurons recorded from the anterior striatum, comprising caudate, putamen and ventral striatum (94 neurons recorded from monkey A and 71 from monkey B). The relationship of striatal neurons, including the 165 neurons reported here, to reward recipient and social actor, and reward inequity has been reported elsewhere^10^,^12^. We analysed neural activity between the onset of the trial end event (black background onset) and 500 ms afterward with a 2-way ANOVA with actor and trial outcome as main factors. We found that 59 neurons (36%) showed differential activity during the trial outcome epoch. These neurons were classified as coding error or correct trials depending on which condition produced the highest neuronal activity. Forty neurons (24%) were classified as signalling an error trial and 19 neurons (12%) were classified as signalling a correct trial.

**Figure 2:**
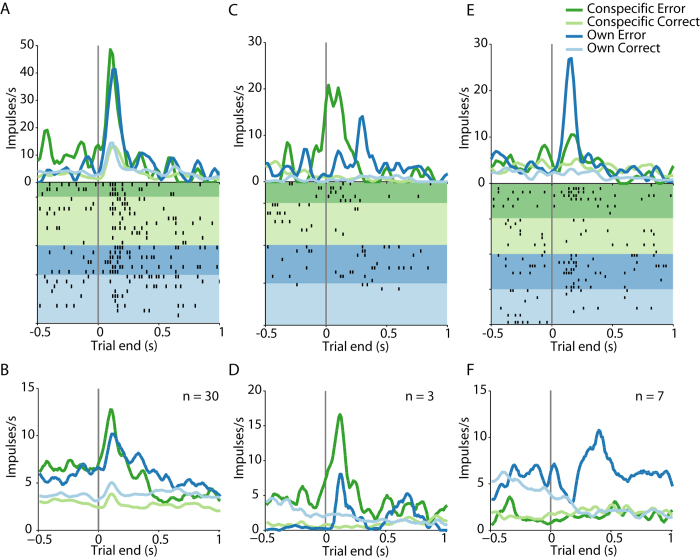
Neurons with performance error activity. (**A,B**) Neuron (**A**) and neuronal population (**B**) with increased activity during errors, regardless of the actor (dark vs. light colors). (**C,D**) Neuron (**C**) and neuronal population (**D**) with increased activity during conspecific’s error. (**E,F**) Neuron (**E**) and population (**F**) with increased activity during own errors. Smoothing filter, σ = 70ms.

Figure 2A shows a neuron with increased activity during error trials, irrespective of the actor (dark colours > light colours). A total of 30 neurons (18%) showed increased activity during errors regardless of the actor who committed the error (Fig. 2B). The apparent differences in activity before the trial ended in error-coding neurons might have reflected the activity of neurons with stimulus-specific expectation activity^13^,^14^. Performance-monitoring neurons were further classified as coding conspecific errors or own errors depending on which condition produced the highest neuronal activity given that the factor ‘actor’ was significant. Figure 2C shows a neuron with increased activity during conspecific errors (dark green) compared to own errors (dark blue), conspecific correct trials (light green), and own correct trials (light blue). Also, three neurons (2%) showed increased activity only during conspecific errors compared to own errors or correct trials (Fig. 2D). Figure 2E shows a neuron signalling own errors (dark blue > dark green or light colours). Additionally, seven neurons (4%) showed increased activity only during own errors (Fig. 2F). The number of neurons that only reflected own errors or only conspecific errors was less than expected by chance. In total, we found 33 neurons (20%) that signalled conspecific errors and 37 neurons (22%) that signalled own errors, while 10 error-signalling neurons (6%) discriminated between actors.

Given that the distribution of errors made in one session was large, we investigated if there was a correlation between the numbers of errors committed by each animal and whether or not a neuron was classified as coding an error. The number of errors committed by either the recorded animal or its conspecific and the type of neuron were not significantly correlated (rho = 0.06, p = 0.44; rho = −0.08, p = 0.29, respectively). Thus, the number of committed errors was not related to classifying a neuron as coding error.

A different subpopulation of striatal neurons showed an increase in activity at the end of a correct trial. A total of 15 neurons (9%) showed increased activity during correct conspecific trials, of which nine neurons differentiated between actors. Similarly, four neurons (2%) showed a differential increase in activity at the end of a correct trial completed by the recorded animal.

#### Anatomical distribution of error-coding neurons

Studies of the human striatum suggest that performance-monitoring might be represented differentially in its different territories^15^,^16^. Therefore, we checked the distribution of error-coding neurons across the different territories of the striatum. We found that the percentage of error-coding neurons in each territory was fairly uniform (26%, 23% and 25%, in caudate, putamen and ventral striatum respectively). Thus, in our data there is no striatal territory specialized in responding to performance errors.

#### Neuronal coding of different error types

During task performance the animals could commit 3 different types of errors (Fig. 1A): not touching the resting key at the start of the trial (ignored trials), releasing the resting key before the onset of the go signal (early key release) and not touching the monitor before the 1.4 s deadline after the onset of the go signal (no touch). We investigated if the activity of the subpopulation of error coding striatal neurons (n = 40) differentiated between error types. Due to the low number of errors committed by each individual (mean = 15), we first analysed neuronal activity at the population level. We compared neuronal activity during the first 500 ms after trial end signal onset following each error type, regardless of who committed the error or if the neuron showed differential activity between actors.

**Figure 3:**
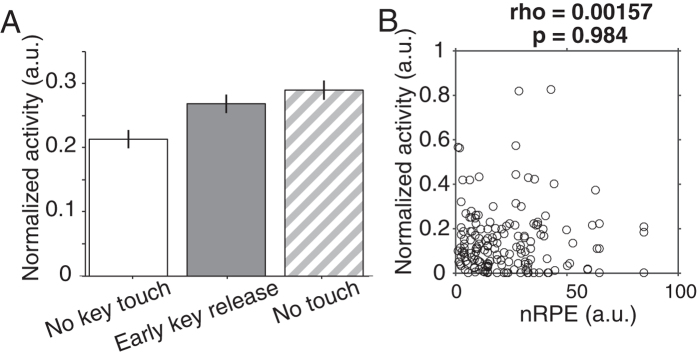
Error-coding neurons discriminate between error types but do not code negative reward prediction errors (nRPE). **(A)** Mean normalized activity of error coding neurons (n = 40) after each different type of error. Activity was significantly increased after early key release or no touch errors compared to no key touch errors (grey and diagonal stripes vs. white; *post-hoc* Holm test after 1-way ANOVA). Error bars show SEM. **(B)** Scatterplot of mean normalized neuronal activity from single error-coding neurons and estimated nRPE.

The effect of error type on neuronal activity on the population of error-related neurons was significant (F(2, 1096) = 6.035, p = 0.002); however, the effect size was small η^2^ = 0.011. We found that the population activity was smaller after ignored trials compared to early key release or no touch errors (Fig. 3A; *post hoc* Holm test, p = 0.0006, and p = 0.01, respectively). However, the difference between early key release errors and no touch errors was not significant (*post hoc* Holm test, p = 0.28). To confirm the generality of this observation, we repeated the same analysis on all single error-coding neurons that were recorded with at least 3 errors of each type. We found that 31% of error-coding neurons (8 out of 22) showed differential activity between different error types. These results suggest that the type of error the animals committed had a small, but significant, effect on the magnitude of the neurons’ response to errors.

#### Error-coding neurons do not reflect reward absence nor negative reward prediction errors

It is possible that error-coding neurons instead of reflecting performance errors actually reflected reward absence because there was no reward delivered after an error. To test this possibility we compared the neuronal activity after a performance error to the activity after a correct trial in which the recorded animal did not expect reward. A large majority (80%, n = 32) of error-coding neurons differentiated between error trials and correct trials without expected reward (Wilcoxon rank-sum, p < 0.05). Furthermore, when we compared the responses to own reward during task performance of the error-neurons (reported elsewhere^10^), we found that 23% (n = 9) also reflected own reward absence. These results suggest that most error coding neurons indeed reflect errors and not reward absence and that largely non-overlapping populations of striatal neurons code own reward or errors. Note that when the animal did not expect reward and none was delivered, by definition, there was no negative or positive reward prediction error.

Performance errors refer to failures to complete a trial, while negative reward prediction errors (nRPE) refer to not receiving a predicted reward. We considered the possibility that performance error activity might be better explained as reflecting nRPE. To test this prediction we trained a TD model based on the animals’ behaviour to generate a proxy for the recorded animal’s own nRPE during each neuronal recording session^17^,^18^. Each error type corresponded to a different epoch during the task. Thus, errors closer to the time of expected reward delivery produced larger nRPE (rho = 0.229, p = 1.36 × 10-21). However, we observed that the correlation between mean firing rate and nRPE was not significant at the population level (Fig. 3B; rho = −0.0015, p = 0.98). This result was further confirmed by the lack of significant correlations between mean firing rate of single error-coding cells (n = 40) and nRPE. Although the activity of error-coding neurons at the population level discriminates between error types, it does not correlate significantly with nRPE.

#### Error-coding neurons do not reflect surprise salience

An alternative explanation to error-coding is surprise salience, i.e. an unexpected event that draws attention. We hypothesized that if a neuron coded surprise salience after an error then it would diminish its activity in subsequent error trials since an error increased the likelihood of a subsequent error, which in turn made the subsequent error less surprising. Therefore, if surprise salience explained activity better than errors, then neuronal activity should correlate with the number of past errors. We found that only 2 out of 40 (5%) error-related neurons showed significant correlation with the number of past errors. At the population level, we observed that the correlation between firing rate and the number of past errors was not significant (rho = −0.036, p = 0.64). These results suggest that the large majority of error-coding activity cannot be explained as reflecting error frequency nor surprise salience.

## Discussion

Here we show that rhesus macaques were sensitive to both their conspecific’s errors and their own errors and that striatal neurons signalled performance errors. The animals made more errors after individually committing an error, but made fewer errors after their conspecific had committed an error. This suggests that past behavioural performance influenced current performance. Most activations of the error-coding striatal neurons responded to conspecific and own errors, although some were actor-specific. Control analyses ruled out that these error responses were related to nRPE or surprise salience. These results suggest that striatal neurons signal performance errors in a social task.

In our task, monkeys’ conditional error rate increased after making their own error but decreased after a conspecific made an error. We discarded the possibility that the presence or absence of own reward on a conspecific’s error trial (i.e. reward for the observer monkey) influenced the observer monkey’s conditional error rate. Our behavioral results, along with results from previous reports^9^,^19^,^20^, suggest that rhesus macaques not only monitor their behavior but also monitor other monkeys’ behavior. The task did not require the animals to show a behaviour consistent with monitoring their conspecific’s performance, in contrast to a previous study^9^. A more explicit behavioural correlate might have shed more light on social error detection. However, in our task the observer monkey needed to prepare to receive a juice reward during some of the conspecific turns and also needed to prepare for its own turn. Thus, to anticipate reward and the start of their turn the observer monkey needed to monitor the other’s performance. Our results suggest that the behaviour of the actor influenced the behaviour of the observer, thus, the observer monitored the actor’s behaviour.

It is unclear by what mechanism the conspecific’s behaviour influenced the observer monkey’s behaviour. One possibility is that the changing reward rate may have motivated the animals to complete the trial following a conspecific’s error. Further testing will allow us to better elucidate the mechanisms behind this behavioural finding.

We observed a decrease in errors in trials with reward for the actor compared to trials without reward. We also observed that both animals made more errors later in a session. Another source of errors were motor inaccuracies, for example not grabbing the key rest at the contact point or touching the screen outside the target area. However, both animals were overtrained on the task for more than 6 months before the first neuronal recordings started, this training time corresponds to performing ca. 20,000 trials. Thus, motor inaccuracies were minimized in behavioural training. In some trials, they could have been distracted by the observer monkey in a non-specific manner. However, both animals remained quiet during the other’s task performance and almost never vocalized in the laboratory. Together, these observations suggest that while we cannot discard in their entirety explanations based on motor performance and/or fluctuations in attention, motivation played an important role in error generation.

The majority of error-coding striatal neurons did not discriminate between actors (30 out of 40) whereas a smaller minority of neurons (10 out of 40) differentiated between actors. Interestingly, it has been observed that neurons in anterior cingulate discriminate between own and conspecific errors^9^. However, some of the conspecific-error neuronal activities in this previous study were better explained by nRPE coding. Importantly, striatal neurons have been shown to code positive RPE^21^,^22^ but very few code nRPE^23^,^24^. In contrast to medial frontal cortex neurons, error-coding striatal neurons seldom reflected nRPE. The lack of a correlation between nRPE and neuronal activity was surprising given that neuronal activity increased for error types closer in time to reward delivery. These data suggest that the performance error signal was independent of nRPE in striatal neurons.

The current data on striatal neurons suggest the existence of a performance monitoring signal outside the prefrontal cortex. Neural activity in the anterior cingulate cortex has long been hypothesized to play a role in detecting self-generated performance errors^2^,^5^. In support of this, single neuron activity in this area has been shown to correlate with performance errors^4^,^6^,^25^. Other cortical areas, including supplementary eye field, frontal eye field and posterior cingulate, also contain neurons that signal performance errors^3^,^25^,^26^,^27^. Our data suggests that the error-related neurons in the striatum may be part of a larger neuronal circuit conveying error signals.

The medial frontal cortex and basal ganglia may also play a role in the generation of error signals^7^,^8^. Error-related activity in the medial frontal cortex has been shown to reliably encode performance errors. This area, including the dorsal anterior cingulate cortex, contains neurons that project to the caudate, putamen and ventral striatum^28^. This error signal could then combine with actor-specific signals found in the striatum^10^ and generate the actor- and error-specific signals we identified in this downstream brain region. In support of this idea, all striatal territories contained similar distributions of error-coding neurons, suggesting a common input. In this scheme, other components of the basal ganglia might contribute towards this error signal. Supporting this idea, a recent study using human intracortical EEG recordings in the internal globus pallidus showed that local field potential in this region generates error-related potentials with shorter latencies than accompanying event-related negativity in cortical EEG^29^. In conclusion, striatal neurons signalling performance errors embedded in a neuronal circuit encompassing the basal ganglia and the prefrontal cortex may play a role in performance monitoring during social interactions.

## Materials and Methods

### Animals

Two adult male *Macaca mulatta* monkeys (animals A and B), weighing 9 kg, served in the study. All experimental procedures were approved by, and performed in accordance with, the University of Cambridge Ethics Committee and the Home Office under the Animals (Scientific Procedures) Act 1986. Animals were implanted with a custom made stainless steel head-restraint devices and recording chambers under general anesthesia.

### Behavioural task

Two monkeys sat opposite each other at a horizontally mounted touch sensitive computer monitor and performed an imperative reward giving task (Fig. 1A,B). In this task, one monkey was the actor, and its conspecific the observer. The actor moved towards a touch screen to complete a trial. The role of actor was indicated by changing half of the monitor surface close to the designated animal from black to gray. To initiate a trial, the actor contacted a touch sensitive resting key placed next to the monitor for 0.5 s. This was followed by the presentation of a reward-predicting cue for 1 s (composed of circles or a square). The actor then waited for a go-signal, released the key and touched the go-signal to receive a juice reward (or none, depending on the payoff) after a 2 s delay. The spectator received a reward (or none) 1 s afterward, which was followed by an inter-trial interval (ITI) indicated by a black screen. ITI varied between 2.5 and 3.5 s. The role of actor and spectator switched after every correct trial. There were no behavioural requirements for the spectator on either task, other than remaining quiet and abstain from disruptive behaviour.

During task performance, the actor could commit three different types of errors (Fig. 1B): (1) not touching the resting key, (2) releasing the resting key before the onset of the go-signal, (3) not touching the touchscreen before the timeout (1.4 s). If the actor committed any of these errors the screen turned black and a timeout of 5 to 7 s (planned trial time + 0.5 s) ensued, the animals did not switch roles, and the trial was reinitiated. The animals did not switch roles until a correct trial was performed. Custom-written software (MATLAB, Mathworks, Natick, MA) running on a Microsoft Windows XP computer controlled task timing.

Rewards consisted of 0.2 mL drops of blackcurrant juice, made from concentrate diluted at a ratio of 1:11 by water (Ribena, GlaxoSmithKline, UK). The animals experienced 4 different reward conditions in a given session: reward to neither, only to the actor, only to the conspecific or to both. In some sessions we only tested: reward only to the actor, only to the conspecific or to both. We pseudo-randomized the sequences of reward conditions that each animal experienced. Liquid delivery was controlled via a solenoid valve (SCB262C068; ASCO, UK).

### Data acquisition

We used conventional electrophysiological techniques to sample extracellular activity from 273 slowly-firing neurons (n = 115, n = 158 in monkeys A and B, respectively) in the anterior striatum of one monkey at a time. We discriminated between slowly-firing neurons and other neurons based on their discharge rates and waveforms^30^. We isolated the activity of single neurons online using a window discriminator (DIS-1, Bak Electronics Ltd., Umatilla, FL), and offline with spike sorting software (Offline Sorter, Plexon Inc., Dallas, TX). The relationship of these neurons to reward recipient and social actor has been reported elsewhere^10^.

### Data analysis

#### Behaviour

To explore the possibility that the conspecific’s error had a behavioral impact on the actor’s behavior we estimated the conditional error rate, that is, the error rate of the observer following a conspecific error or following an own error while ignoring the conspecific’s correction trial. Higher conditional error rates suggest that a conspecific’s (or own) errors increased the probability of an observer error.

To further explore the effect of own and conspecific errors in error probability we used a logistic regression model with terms for each combination of error and actor. The model, thus, contained two terms plus offset:


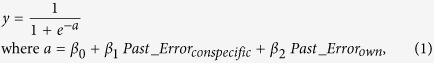


The terms *Past_Error*_*conspecific*_ and *Past_Error*_*own*_ took a value of 1if the corresponding animal made an error in the previous trial (excluding the correction trial) and 0 otherwise. Finally, *y* was the conditional error rate of the animal.

To address any possible dependence on the sequence of each animal’s choices (error vs correct trials) we estimated the confidence intervals of the odds ratio for each predictor using bootstrap. This method eliminates the temporal dependence on the animal’s choices by sampling with replacement from every case. The 95% confidence intervals were established from a bootstrap distribution of odds ratio values with 2,000 iterations.

To take into account the effect of own reward presence during conspecific errors we included a term related to reward history to equation (1):


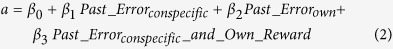


where *Past_Error*_*conspecific*_*_and_Own_Reward* took the value of 1 if the observer would have received a reward in the previous trial when the conspecific made an error and 0 otherwise.

We used the Wald t-test to assess whether the contribution of each predictor was significant in each model.

#### Neurons

To analyse the neuronal response to behavioral errors we contrasted the neuronal response during the first 500 ms after the onset of the trial end black screen between error and correct trials and between actors (own vs. conspecific). We used a 2-way ANOVA with factors ‘actor’ (conspecific/own) and ‘trial outcome’ (error/correct) to this end. We aided our classification by discerning which factor generated the highest average activity.

We classified neuronal activity following these rules. If factor ‘trial outcome’ was significant (*p* < 0.05) then the neuron was classified as coding error trial if the mean activity was higher after an error trial compared to correct trials. Similarly, if factor ‘actor’ was significant (*p* < 0.05), a neuron was classified as coding own trial if its mean activity was higher during own trials compared to conspecific trials. Finally, if both factors were significant, then the neuron was classified as coding trial outcome and actor.

We normalized neuronal activity by subtracting the firing rate of each trial to the maximum firing rate of all trials and dividing by the maximum firing rate of all trials. With this normalization method, all neuronal activity is bound in the [0, 1] interval.

#### Temporal difference modeling

Neuronal responses to performance errors might be better explained as reflecting negative reward prediction errors (nRPE). Performance errors refer to failures to complete a trial while negative reward prediction errors (nRPE) refer to not receiving a predicted reward. Because the task included trials when the recorded animal expected both, reward and no reward, we could test this hypothesis as follows. First, we obtained the error rate of each animal and each error type on all recording sessions. Second, we trained a temporal difference (TD) model with these error rates, a learning rate of 0.2 and without temporal discounting^17^,^18^. Third, we used the estimated weights to establish a TD model for every neuronal recording session. We only used trials when the recorded animal was rewarded. Fourth, we obtained the Spearman’s rank correlation between the firing rate of each neuron and the error-related activity to the magnitude of the estimated nRPE obtained with the TD model. Since we were only interested in the relationship between neuronal activity and nRPE or performance errors, we did not explore the correlation between positive reward prediction errors and neuronal activity.

All statistical tests on behavioural and neuronal data were two-tailed unless otherwise noted. All analyses were performed in MATLAB (Mathworks, Natick, MA).

## Additional Information

**How to cite this article**: Báez-Mendoza, R. and Schultz, W. Performance error-related activity in monkey striatum during social interactions. *Sci. Rep.* **6**, 37199; doi: 10.1038/srep37199 (2016).

**Publisher’s note**: Springer Nature remains neutral with regard to jurisdictional claims in published maps and institutional affiliations.
